# Death during Chloroform Inhalation

**Published:** 1882-01

**Authors:** 


### ARTICLE III.

### *Death During Chloroform Inhalation.*

On the 20th of December a man named E. H. Tapper, from Hammond, Indiana, called at the dental office of Sovereign Brothers, at No. 107 Clark Street, Chicago, to have some teeth extracted. He seemed to be under the influence of liquor, and the dentists demurred to giving him chloroform, but he insisted upon the operation being performed, and finally the dentist sent for Dr. Lewis Dodge to administer the chloroform, while Tapper went out after a friend, Mr. F. G. Dannecker, an attorney, to be a "witness to the bargain," as he said. The drug was administered, and, when in a proper state of insensibility, the dentist began extracting the teeth. He suddenly stopped when he saw the man didn't look right, and efforts were made to resuscitate him, but the man was a corpse.

The inquest was held on December 28th in the coroner's office. The jury being present and sworn, the examination of witnesses began. Mr. Dannecker, the friend of the deceased, was present, and in a great measure conducted the examinations.

The first witness called was Dr. Lewis Dodge, who resides at No. 219 East Ohio Street; had practiced medicine 35 years; graduated from the Homeopathic Medical College, of Philadelphia; had his diploma and a certificate from the State Board of health—the last produced in court; was called on to administer chloroform on the 20th of December, about 3 p. m., in the office of Sovereign Brothers; had been called there several times previously; found on the last visit to the office the deceased; examined over his chest to see if there were any warnings against it; the inference was that deceased wanted it; witness placed his ear carefully over chest of deceased and examined his pulse; the examination lasted perhaps, two or three minutes, as usual in such cases; he caused the vest to be unloosed so there would be no obstruction to respiration; had administered chloroform a great many times. The bottle and cup were produced here, and the Doctor explained to the jury the mode of applying it. Perhaps half an ounce was given; the patient was considerably excited, and it took longer for the chloroform to take effect; this time it required perhaps half an hour; there was nothing else unusual in the operation; they were guided by the effects and not by the quantity inhaled; he had been singing in the German language perhaps half a minute before he died; there was nothing unusual in his appearance; could not say how many teeth had been extracted; there was no struggle, but a gradual sinking observed by the dentist and myself; means were immediately taken for resuscitation; was perfectly familiar with the methods of resuscitation; it could not have been more than two minutes after the fainting before he died; first raised up the body in the chair and rotated it; then laid him down on the floor with his head lower than his body; requested one of the Sovereign brothers to call Dr. Dyas, who had his office on the same floor; Dr. Dyas came in and helped attempt artificial respiration, as described in the books; this was continued ten or twenty minutes, even after the symptoms were that life had departed; ammonia was applied to his nostrils and a cloth wet with alcohol placed over his heart; he was also chafed with alcohol over the chest and abdomen; his bare feet were slapped with a wet towel, also his forehead and face; did not pull his tongue out, because he thought it a waste of time, as other things were more important; there had been very little hemorrhage from the teeth, not enough to make its way to the throat.

After he was thoroughly satisfied that life was extinct witness telephoned for the Coroner; in the chair the patient was reclining rather more than is usual in extracting teeth; his head was as far back as the chair would admit, very near a horizontal position; never hastened the inhalation; the window was opened to admit some fresh air; was also interested in a real estate office, but paid little attention to it; the patient remarked several times, "Feel my pulse, Doctor; don't kill me;" this is very common and is not regarded as serious.

Mr. F. G. Dannecker here asked witness if he remembered that he advised against the administration of chloroform, and the Doctor replied that he did. The first inhalation of chloroform usually quickens the pulse, but after that there is a little depression; the pulsation continued after witness had ceased to give deceased chloroform; there was nothing unusual in his appearance; the pulse was about as usual up to the time when he turned him over to the dentist; when deaths occur from syncope it is like a stroke of lightning caused by a stoppage of the heart.

In answer to a question from Dr. DeWolf, witness said the patient's condition would be considered by himself dangerous, and he should never administer chloroform again to a man when he had premonitions of danger; witness said the man seemed most unusually excited; could not say that he had his finger on the pulse when it stopped; the man made several motions with his right leg, as if pushing away something; witness' opinion was that the man died from paralysis of the heart; this was the first fatal case he ever had; had used it in over a thousand cases with no ill effects; many times patients were in as excitable a condition as in this case; the chloroform was of the usual strength and pure.

Dr. S. J. Sovereign was next called; was a dentist; had been in the business of a dentist for fifteen years; was a graduate of the Canadian Royal College of Dental Surgeons; on Tuesday, December 20, about 3 P. M., Mr. Tapper came in his office and wanted chloroform administered and teeth extracted; witness refused to apply it; Tapper refused to have his teeth out without the drug, and wanted a physician sent for; witness sent for Dr. Dodge and Tapper returned with Mr. F. G. Dannecker, an attorney; Tapper took a seat in the chair, the Doctor examined him and gave him the chloroform; witness introduced Tapper to Dr. Dodge, and they laughed and joked together; after the chloroform had been administered witness drew a few teeth from his lower jaw, and stopped when he saw the appearance of the deceased was not favorable; the chair is so fixed that the hips can be raised higher than the head; the Doctor then raised him up and the patient was rotated in accordance with the directions of the Doctor; the treatment was continued some twenty minutes, when Dr. Dyas said life was extinct. Tapper said his object in having Dannecker there was to have a witness to the bargain; witness thought if the man was not in proper condition it was the Doctor's business to say so; did not detect any signs of intoxication upon Tapper; it was a common thing for ladies to make their wills before taking chloroform for the purpose of having teeth extracted; when the teeth were extracted he was raised from a reclining position; his teeth were in a bad state; some blood came from them but not much.

Dr. A. E. Small was now called. Had known Dr. Dodge over thirty years; knew of Dr. Dcdge's graduation from the college in Philadelphia; had known him nearly all the time since; believed the treatment in this case was rational and proper; might have added something to the treatment; Dr.

Dodge's standing was good; would not administer chloroform to a man under the influence of liquor; persons about to have teeth extracted would many times take a glass of brandy or whiskey to keep their courage up; in such cases he had administered it, but never when a man was under the influence.

Charles W. Sovereign, brother of the other dentist, was sworn. Knew nothing of the case until after the teeth were extracted; witness described the means adopted for resuscitation in the same terms as previous witnesses; Dr. Dodge had administered chloroform in his office over twenty times, and never had any trouble; considered the Doctor a very careful man; saw no signs of intoxication in Tapper.

Dr. S. J. Sovereign was recalled. Had had several doctors to administer chloroform; the usual price paid the doctors was from $2 to $4; paid Dr. Dodge $2; always wanted patients to bring their own physicians; had called in Drs. Reynold, Wright, Dyas, and others; had employed Dr. Dodge in his own family.

Dr. Oscar C. DeWolf, Health Commissioner, physician and surgeon of twenty-two years standing, was called. The administration of chloroform in dental practice was universally condemned; the elevating the patient into the upright condition was the dangerous and fatal mistake; as anæsthetics and comparatively not dangerous, there were ether and nitrous oxide. In an attempt to recover an individual from that condition the efforts should have been systematic and efficient; this he thought was not done in this case; he thought the tongue should have been pulled forward; the patient should have been placed in a reverse position with his head down; if blood got in the windpipe it would stop respiration; this was not a fatal dose of chloroform if there was plenty of atmospheric air admitted; the cup could not be applied close enough to exclude air unless the cloth should exclude it; under certain circumstances there was no impropriety in giving a man a moderate dose of alcohol before a dose of chloroform, but not to intoxication; should hesitate to administer chloroform for thirty minutes; an expert physician would not require more than three minutes to examine the heart; saw nothing in the testimony indicating a lack of atmospheric air; he said the universal testimony of competent physicians totally forbids the administration of chloroform in dental practice; had heard of deaths from ether; the difference between dental practice and others was the difference in position; the upright position was terribly dangerous.

Dr. Theo. J. Bluthardt, County Physician, of twenty years practice, was sworn. The administration of chloroform was always at the risk of the patients. It was frequently done, but he would prefer other anæsthetics; his impression was that the operators were frightened or were unskillful; did not believe in holding ammonia under the nose of a person who had ceased to breathe, and thought that rubbing with alcohol was not enough to stimulate the heart; had no evidence that this man was drunk; pulling out the tongue might have a good effect.

The post-mortem of Dr. T. J. Bluthardt is of considerable length, and concludes as follows:

"1. That death occurred from suffocation caused by pulmonary congestion and paralysis of the heart.

"2. That the direct cause was the use of chloroform."

Dr T. C. Duncan, editor of the *Medical Journal*, had a good opinion of both Dr. Dodge and Dr. Dyas; from the post-mortem examination it was shown that the man had a weakened heart; the question of the strength of chloroform was important; physicians really hesitated to give chloroform in dental cases; preferred nitrous oxide; the methods employed in resuscitating the patient were those usually employed; there are other means, and patients die under them all; artificial respiration should be kept up for some time, but when so able a physician as Dr. Dyas pronounced a man dead, he should not hesitate to believe him; the man evidently died of paralysis of the heart; it was common to give stimulants with anæsthetics of all kinds; physicians had their preferences for resuscitation according as they got used to it; any anæsthetic is risky, but where a person is timid about pain he always watched the patient very carefully; it was quite common to hear frightened remarks about patients in such cases; it was the duty of a physician to consider the condition of a patient, but when a patient was excited chloroform generally calmed him; would not hesitate to administer chloroform for thirity minutes or much longer; some went under the influence much easier than others.

Dr. Robert L. Rea was next sworn. Had been practicign thirty years; gave his method of giving chloroform; preferred an empty stomach, and gave from two to four teaspoonfuls of either whiskey or brandy ; always preferred to give ether, beginning slowly, and allowing air to enter freely; constantly kept his finger on the pulse; wanted to insure the falling forward of the tongue, and so always pulling the tongue forward and the lower jaw at the same time; never had any serious results: all anæsthetics are dangerous, even nitrous-oxide; no phsyical examination will denote whether it is safe or not for any patient; in a case of paralysis of the heart it would make but very little difference what was done, but everything possible should be done in any case; in this one he should have done some things differently; dentists should be required to keep an electric battery in their offices ready for use; a physician should always be called on to decide whether it was safe to administer chloroform or not; this could be decided in a minute or two; an overdose of liquor would add to the effect of the chloroform; would not administer for thirty minutes under any circumstances for fear of a cumulative effect.

Dr R. G. Bogue was called. He said he never would give chloroform to any patient where the head was to be raised above a horizontal position after insensibility; thought the treatment availed very little after the patient came into an appearance of apparent dying; gave his opinion of resuscitation, which did not differ materially from those of previous witnesses.

Dr. Walter W. Allport was called. He regarded the operation of extracting teeth as not of sufficient consequence to warrant the administration of chloroform; was familiar with the use of anæsthetics; never used it to exceed fifteen minutes; if chloroform is administered very slowly, and atmospheric air allowed to enter, it might not be dangerous to apply it for thirty minutes; it is better to have it take effect as soon as possible and avoid cumulative effects; thought the methods employed in this case for resuscitation were hardly sufficient; efforts should be kept up for half or three-fourths of an hour, and require very prompt and energetic action; statistics show that the deaths reported from ether are about 1 in 23,000 ; from chloroform 1 in 3,000; from both ether and chloroform 1 in 5,000.

Dr. S. J. Sovereign was recalled. He said no physician ever told him that it was dangerous to raise a patient when under the influence of chloroform.

The jury then retired, and after an absence of nearly two hours returned with the following verdict:

That Edgar H. Tapper came to his death December 20th, at the office of Sovereign Bros., No. 107 Clark Street, from suffocation caused by pulmonary congestion and paralysis of the heart, caused by the inhalation of chloroform administered by Dr. Lewis Dodge for the purpose of having his teeth extracted at the said dental office, and we, the jury, deprecate the use of chloroform in such cases. That we do not consider that, with the known danger of administering chloroform, sufficient precautions were observed, and that the efforts for his restoration should have been more vigorous and protracted, and will suggest that dentists should be compelled to keep all proper appliances for resuscitating patients using anæsthetics in their offices.

